# Refractory Post‐TIPS Hepatic Encephalopathy Complicated by Small Bowel Obstruction: A Therapeutic Dilemma

**DOI:** 10.1155/crgm/3799217

**Published:** 2026-09-27

**Authors:** Ammar Ismail, Amira Alnakeb, Saba Altarawneh, Joseph Depasquale

**Affiliations:** ^1^ Jersey City Medical Center, Rutgers University, New Brunswick, New Jersey, USA, rutgers.edu; ^2^ Faculty of Medicine, Mansoura University, Mansoura, Egypt, mans.edu.eg

## Abstract

**Introduction:**

Refractory hyperammonemic encephalopathy in cirrhosis presents a therapeutic challenge, particularly in post‐TIPS patients or with contraindications to standard therapies. We report a fatal case complicated by small bowel obstruction (SBO), highlighting the limitations of current management strategies.

**Case Presentation:**

A 69‐year‐old male with decompensated NASH cirrhosis and recent TIPS revision for shunt stenosis developed SBO. He presented with severe encephalopathy (GCS 5), seizures, and MRI‐confirmed cerebral edema. Due to SBO, lactulose and rifaximin were not effective. Despite aggressive dialysis with continuous venovenous hemodialysis (CVVHD) reducing ammonia from 447 to 160 μmol/L, he progressed to irreversible cerebral edema and met brain death criteria.

**Discussion:**

Post‐TIPS hyperammonemia carries high mortality, especially when compounded by SBO. CVVHD effectively lowers ammonia but may fail to prevent neurologic injury if initiated late.

**Conclusion:**

This case underscores the need for proactive RRT and novel ammonia‐lowering strategies in high‐risk patients, especially when gut decontamination is not feasible.

## 1. Introduction

Hepatic encephalopathy (HE) is a devastating complication of cirrhosis, affecting 30%–40% of patients during their disease course [1, 2]. Post‐transjugular intrahepatic portosystemic shunt (TIPS) HE is particularly severe, with hyperammonemia driving neurologic decline and mortality [3–5]. While lactulose and rifaximin are first‐line therapies [5], their administration is limited in intestinal obstruction or refractory cases similar to our case.

Hyperammonemia exceeding 200 μmol/L poses a critical risk for cerebral edema and irreversible injury [6]. Renal replacement therapy (RRT), especially continuous modalities like continuous venovenous hemodialysis (CVVHD), may mitigate this risk by providing sustained ammonia clearance [7, 8]. However, evidence remains sparse, particularly in post‐TIPS patients with concurrent small bowel obstruction (SBO).

We present a fatal case of refractory hyperammonemia post‐TIPS, complicated by SBO, to highlight the therapeutic dilemma of managing HE when standard therapies fail, the role of CVVHD in ammonia clearance, and the poor prognosis despite biochemical improvement.

## 2. Case Presentation

A 69‐year‐old man with a history of decompensated NASH‐related cirrhosis (recurrent ascites, HE, nonbleeding varices s/*p* banding), portal vein thrombosis (PVT) with superior mesenteric vein (SMV) extension status post‐TIPS and thrombectomy (with recent TIPS revision to 10 mm due to stenosis 2 days prior to admission), hypertension, diabetes, and a recurrent incarcerated right inguinal hernia was brought to the emergency department by his family for acute‐onset altered mental status. The patient was initially nonverbal, unresponsive to commands, and progressively deteriorated to a Glasgow Coma Scale (GCS) of 5, necessitating intubation and ICU admission.

Initial management focused on HE with lactulose and rifaximin; however, his mental status continued to decline. On ICU Day 2, new twitching of the hands and eyelids was observed, and video EEG confirmed seizure activity, prompting initiation of IV levetiracetam, propofol infusion, and midazolam infusion for status epilepticus. Concurrently, CT of the abdomen and pelvis demonstrated SBO with a transition point within the right inguinal hernia sac (Figure 1). Because nasogastric output was initially low and the abdomen was scaphoid without significant distension, conservative management was pursued given the patient’s prohibitive surgical risk. Lactulose was continued through the nasogastric tube and rectally, while rifaximin was administered through the nasogastric tube. Three days after the CT findings, nasogastric output increased to > 1500 mL/24 h and ammonia levels rose sharply from 264 μmol/L to over 447 μmol/L. Lactulose and rifaximin were therefore discontinued, and RRT was initiated. The patient could not tolerate intermittent hemodialysis (HD) because of high vasopressor requirements and was transitioned to CVVHD.

**FIGURE 1:**
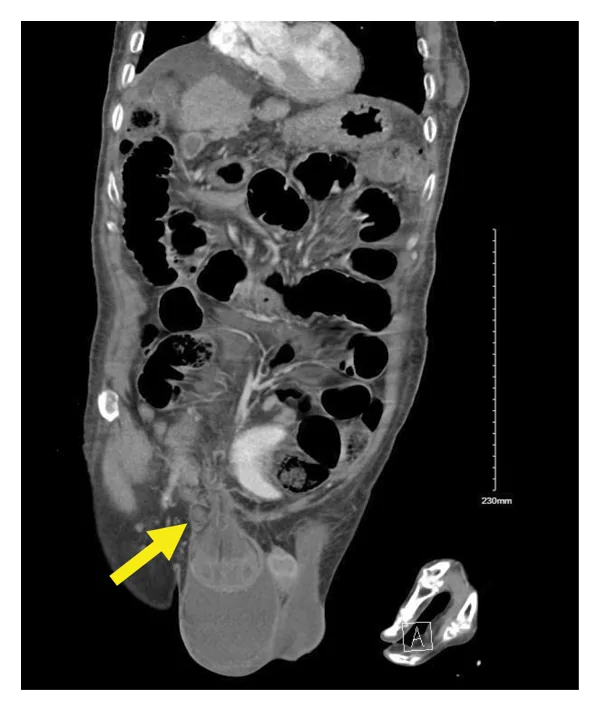
Coronal contrast‐enhanced CT image of the abdomen and pelvis showing dilated small bowel loops with transition point at inguinal hernia sac (arrow).

Because the patient had recently undergone TIPS revision with dilation of the stenotic segment just 2 days prior to presentation, there was early consideration for possible TIPS occlusion in light of worsening hyperammonemia and encephalopathy. As the original procedure was performed externally, TIPS occlusion was deferred to the managing interventional team familiar with the patient’s prior intervention. Plans were initiated to transfer the patient back to the facility where the TIPS procedure had been performed for evaluation and potential TIPS occlusion. However, transfer was delayed and ultimately not possible due to the patient’s hemodynamic instability.

CVVHD was performed for a total period of around 60 h, and although CVVHD was transiently interrupted several times for a total period of around 8 h, to obtain a brain MRI, and for technical issues like filter clotting, ammonia levels dropped from 447 μmol/L to 160 μmol/L over the total period. Despite this, his neurologic status did not improve. Sedation was held for > 48 h, yet brainstem reflexes remained absent. MRI brain, which was performed around 12 h after starting CVVHD, revealed diffuse cerebral edema consistent with severe encephalopathy. EEG demonstrated no cortical activity for a period of 48 h after brain MRI findings. After extensive family discussions regarding his poor neurologic prognosis, care was transitioned to comfort measures, and the patient died shortly after extubation.

## 3. Discussion

HE is one of the most debilitating complications of cirrhosis, severely impacting patients’ quality of life and healthcare resource utilization [1]. It marks the transition to decompensated disease, akin to ascites or variceal bleeding [9]. The prevalence of overt hepatic encephalopathy (OHE) ranges from 10%–14% at cirrhosis diagnosis [10, 11] to 16%–21% in decompensated cases [9, 12] and surges to 10%–50% post‐TIPS [3, 4]. Cumulatively, 30%–40% of cirrhotic patients experience OHE during their disease course, often recurrently [2], while minimal or covert hepatic encephalopathy (CHE) affects 20%–80% [13]. Post‐TIPS HE is particularly challenging, as excessive shunt flow due to large stent diameter can precipitate intractable symptoms [5].

SBO represents a clinically challenging scenario that has received limited attention in the literature, particularly when complicated by OHE. Because first‐line therapies for OHE are generally administered enterally rather than intravenously, patients with concurrent SBO may require alternative pharmacologic or nonpharmacologic management strategies. In addition, SBO may itself exacerbate HE. Intestinal stasis can promote bacterial overgrowth and gut microbial alteration, while prolonged contact between luminal contents and the intestinal mucosa may increase ammonia absorption. Disruption of the intestinal barrier may further enhance permeability, bacterial translocation, and portal endotoxemia, thereby amplifying systemic inflammation and contributing to worsening encephalopathy [14–16].

Current guidelines recommend lactulose and rifaximin as first‐line therapies [5], yet evidence suggests they offer no prophylactic benefit against post‐TIPS HE compared to placebo [17]. Alternative pharmacologic therapeutic options include high‐volume polyethylene glycol (PEG) 3350, which may provide greater clinical efficacy than enteral lactulose; however, its use is also limited to enteral administration [18, 19]. Off‐label metronidazole may be considered as an alternative to rifaximin; however, evidence supporting its use remains limited. One study demonstrating no significant differences between the two agents in clinical outcomes, length of hospital stay, or reduction in serum ammonia levels [20]. For patients with Grade 3–4 HE who are unable to swallow or are at high risk of aspiration, rectal administration of lactulose enemas represents another therapeutic option [19]. In refractory cases, shunt reduction or TIPS occlusion may reverse HE [21].

HD emerges as a potential salvage therapy, although the literature is limited to isolated case reports. For instance, Kaneda et al. described a cirrhotic patient with chronic kidney disease whose recurrent HE resolved with daily HD but recurred when frequency was reduced [22]. Similarly, Viguera et al. reported a noncirrhotic patient with hyperammonemia (445 μmol/L) and seizures in whom ammonia levels improved with CVVHD, but clinical outcomes were not reported [23]. These cases underscore HD’s role in ammonia clearance, particularly when levels exceed 150–200 μmol/L, a threshold linked to intracranial hypertension [6].

Intermittent HD is highly effective at rapidly clearing ammonia, making it the preferred modality in many hyperammonemic crises. This is particularly important given that prolonged hyperammonemia increases the risk of irreversible neurologic damage [7, 24]. However, the rapid solute and fluid shifts associated with intermittent HD may exacerbate cerebral edema, a critical concern in patients with severe HE or acute liver failure [25–27].

In contrast, CVVHD offers a more gradual ammonia clearance due to its lower blood flow rates (Qb) compared to intermittent HD. While this might suggest reduced efficacy, CVVHD provides the advantage of sustained ammonia removal with fewer treatment interruptions [7]. More importantly, its slower fluid and solute shifts minimize the risk of worsening intracranial hypertension, making it a safer option in hemodynamically unstable patients or those at high risk of cerebral edema [26, 27].

Standard CVVHD is primarily designed to remove water‐soluble, non–protein‐bound solutes [28]. Ammonia, which is water‐soluble and not protein bound so can effectively be cleared by CVVHD; however, there are additional mediators implicated in the pathophysiology of HE including inflammatory cytokines, such as TNF‐α, IL‐6, and IL‐17, along with bile acids and trace elements (e.g., manganese) [29]. Manganese may be effectively cleared by CVVHD [30], but inflammatory cytokines (TNF‐α, IL‐6, and IL‐17) have large molecular sizes that limit their efficient clearance, and bile acids are extensively bound to albumin and therefore are not effectively removed [28, 31].

Although brain MRI performed 12 h after the initiation of CVVHD revealed signs of cerebral edema, serum ammonia levels decreased significantly from 447 μmol/L to 160 μmol/L over a 60‐h period (Figure 2). Despite this marked biochemical improvement, there was no corresponding change in neurologic function. As shown in Figure 2, ammonia levels declined more consistently during the period of relatively uninterrupted CVVHD between hours 37 and 60. In contrast, periods with more frequent CVVHD interruptions coincided with greater fluctuations in serum ammonia levels. These findings suggest that minimizing interruptions in CVVHD may promote more sustained ammonia clearance.

**FIGURE 2:**
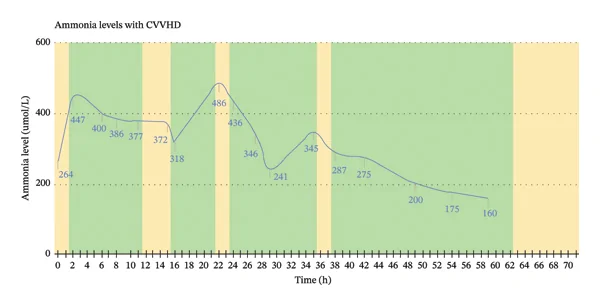
Trend of ammonia levels in relation to CVVHD therapy. Green‐shaded regions indicate time intervals when CVVHD was active, while orange‐shaded regions denote interruptions in therapy.

Studies have demonstrated that CVVHD achieves effective ammonia clearance, particularly when higher ultrafiltration rates are employed [8]. Thus, while intermittent HD remains a choice for rapid ammonia reduction in stable patients, CVVHD may be preferable in critically ill or unstable individuals, where its gentler hemodynamic profile and continuous clearance might offer a therapeutic advantage.

## 4. Conclusion

This case illustrates the lethal synergy of post‐TIPS hyperammonemia and SBO, where standard HE therapies were precluded and CVVHD, though biochemically effective, failed to reverse neurologic injury. SBO played a significant role in clinical deterioration because of inability to administer first‐line medications and worsening encephalopathy due to stasis and bacterial overgrowth. Relative delay in RRT initiation may make ammonia‐lowering treatments ineffective, as cerebral edema becomes irreversible. While CVVHD’s gradual clearance is safer in unstable patients, its interrupted use (e.g., for imaging) may compromise efficacy. Future studies should define the optimal indications and timing for RRT in patients with severe hyperammonemia including post‐TIPS HE who cannot receive conventional enteral therapy (e.g., intestinal obstruction).

## Author Contributions

Ammar Ismail and Amira Alnakeb contributed to data collection, literature review, and drafting of the manuscript. Saba Altarawneh and Joseph Depasquale supervised the preparation of the case report and critically reviewed and edited the manuscript.

## Funding

Article processing charges are to be covered by Rutgers University.

## Disclosure

All authors reviewed and approved the final version of the manuscript.

## Consent

Written consent has been obtained from the patient’s family member given that patient expired.

## Conflicts of Interest

The authors declare no conflicts of interest.

## Data Availability

Data sharing is not applicable to this article as no datasets were generated or analyzed during the current study.

## References

[1] Rakoski M. O. , McCammon R. J. , Piette J. D. et al., Burden of Cirrhosis on Older Americans and Their Families: Analysis of the Health and Retirement Study, Hepatology. (2012) 55, no. 1, 184–191, 10.1002/hep.24616. PMC3462487 21858847

[2] Amodio P. , Del Piccolo F. , Pettenò E. et al., Prevalence and Prognostic Value of Quantified Electroencephalogram (EEG) Alterations in Cirrhotic Patients, Journal of Hepatology. (2001) 35, no. 1, 37–45, 10.1016/s0168-8278(01)00129-5. 11495040

[3] Papatheodoridis G. V. , Goulis J. , Leandro G. , Patch D. , and Burroughs A. K. , Transjugular Intrahepatic Portosystemic Shunt Compared With Endoscopic Treatment for Prevention of Variceal Rebleeding: A Meta-Analysis, Hepatology. (1999) 30, no. 3, 612–622, 10.1002/hep.510300316. 10462365

[4] Nolte W. , Wiltfang J. , and Schindler C. , Portosystemic Hepatic Encephalopathy After Transjugular Intrahepatic Portosystemic Shunt in Patients With Cirrhosis, Hepatology. (1998) 28, no. 5, 1215–1225. 10.1002/hep.510280508 9794904

[5] Vilstrup H. , Amodio P. , and Bajaj J. , Hepatic Encephalopathy in Chronic Liver Disease: 2014 Practice Guideline by AASLD and EASL, Hepatology. (2014) 60, no. 2, 715–735. 10.1002/hep.27210 25042402

[6] Bernal W. and Wendon J. , Acute Liver Failure, New England Journal of Medicine. (2013) 369, no. 26, 2525–2534, 10.1056/nejmra1208937. 24369077

[7] Gupta S. , Fenves A. Z. , and Hootkins R. , The Role of RRT in Hyperammonemic Patients, Clinical Journal of the American Society of Nephrology. (2016) 11, no. 10, 1872–1878, 10.2215/cjn.01320216. PMC5053785 27197910

[8] Slack A. J. , Auzinger G. , Willars C. et al., Ammonia Clearance With Haemofiltration in Adults With Liver Disease, Liver International. (2014) 34, no. 1, 42–48, 10.1111/liv.12221. 23786538

[9] D’Amico G. , Morabito A. , Pagliaro L. , and Marubini E. , Survival and Prognostic Indicators in Compensated and Decompensated Cirrhosis, Digestive Diseases and Sciences. (1986) 31, no. 5, 468–475, 10.1007/bf01320309. 3009109

[10] Saunders J. B. , Walters D. J. R. , and Ap P. A. , A 20-Year Prospective Study of Cirrhosis, British Medical Journal Clinical Research Edition. (1981) 282, no. 6260, 263–266. 10.1136/bmj.282.6260.263 PMC1504019 6779978

[11] Jepsen P. , Ott P. , Andersen P. K. , Sørensen H. T. , and Vilstrup H. , Clinical Course of Alcoholic Liver Cirrhosis: a Danish Population-Based Cohort Study, Hepatology. (2010) 51, no. 5, 1675–1682, 10.1002/hep.23500. 20186844

[12] Coltorti M. , Del Vecchio-Blanco C. , and Caporaso N. , Liver Cirrhosis in Italy. A Multicentre Study on Presenting Modalities and the Impact on Health Care Resources, Italian Journal of Gastroenterology. (1991) 23, no. 1, 42–48. 1747499

[13] Romero-Gómez M. , Córdoba J. , Jover R. et al., Value of the Critical Flicker Frequency in Patients With Minimal Hepatic Encephalopathy, Hepatology. (2007) 45, no. 4, 879–885, 10.1002/hep.21586. 17393525

[14] Mo J. , Gao L. , Zhang N. et al., Structural and Quantitative Alterations of Gut Microbiota in Experimental Small Bowel Obstruction, Public Library of Science ONE. (2021) 16, no. 8, 10.1371/journal.pone.0255651. PMC8336877 34347831

[15] Patidar K. R. and Bajaj J. S. , Covert and Overt Hepatic Encephalopathy: Diagnosis and Management, Clin Gastroenterol Hepatol Off Clin Pract Journal Am Gastroenterol Assoc. (2015) 13, no. 12, 2048–2061, 10.1016/j.cgh.2015.06.039. PMC4618040 26164219

[16] Feng X. , Li X. , Zhang X. et al., Hepatic Encephalopathy in Cirrhotic Patients and Risk of Small Intestinal Bacterial Overgrowth: A Systematic Review and Meta-Analysis, BioMed Research International. (2022) 2022, no. 1, 10.1155/2022/2469513. PMC9596239 36303585

[17] Riggio O. , Masini A. , and Efrati C. , Pharmacological Prophylaxis of Hepatic Encephalopathy After TIPS, Journal of Hepatology. (2005) 42, no. 5, 674–679. 10.1016/j.jhep.2004.12.028 15826716

[18] Li M. , Zhang Z. , Chen Q. , Zhou X. , Shui D. , and Huang J. , Comparative Effectiveness and Safety of Polyethylene Glycol Electrolyte Solution Versus Lactulose for Treatment of Hepatic Encephalopathy: A Systematic Review and Meta-Analysis, Journal of Clinical Gastroenterology. (2022) 56, no. 1, 41–48, 10.1097/MCG.0000000000001621. PMC8647701 34739404

[19] Bajaj J. S. , Jakab S. S. , Jesudian A. B. et al., ACG Clinical Guideline: Hepatic Encephalopathy, American Journal of Gastroenterology. (2026) 121, no. 3, 588–618, 10.14309/ajg.0000000000003899. 41773757

[20] Mekky M. A. , Riad A. R. , Gaber M. A. , Abdel-Malek M. O. , and Swifee Y. M. , Rifaximin Versus Metronidazole in Management of Acute Episode of Hepatic Encephalopathy: An Open Labeled Randomized Clinical Trial, Arab Journal Gastroenterol Off Publ Pan-Arab Assoc Gastroenterol. (2018) 19, no. 2, 76–79, 10.1016/j.ajg.2018.06.001. 29935863

[21] Fanelli F. , Salvatori F. M. , and Rabuffi P. , Management of Refractory Hepatic Encephalopathy After TIPS, American Journal of Roentgenology. (2009) 193, no. 6, 1696–1702. 10.2214/AJR.09.2968 19933667

[22] Kaneda H. , Haruyama T. , Chiba S. et al., A Patient With Recurrent Hepatic Encephalopathy Treated With Long-Term Hemodialysis, Tohoku Journal of Experimental Medicine. (1977) 123, no. 3, 227–234, 10.1620/tjem.123.227. 601771

[23] C V. A. , Ab S. , Da S. , and Nb G. , Late-Onset Multiple Acyl-CoA Dehydrogenase Deficiency, 2025, The Neurohospitalist, 10.1177/19418744251324959. PMC11881097 40051723

[24] Lévesque R. , Leblanc M. , Cardinal J. , Teitlebaum J. , Skrobik Y. , and Lebrun M. , Haemodialysis for Severe Hyperammonaemic Coma Complicating Urinary Diversions, Nephrology Dialysis Transplantation. (1999) 14, no. 2, 458–461, 10.1093/ndt/14.2.458. 10069214

[25] Warrillow S. J. and Bellomo R. , Preventing Cerebral Oedema in Acute Liver Failure: The Case for quadruple-H Therapy, Anaesthesia & Intensive Care. (2014) 42, no. 1, 78–88, 10.1177/0310057x1404200114. 24471667

[26] Davenport A. , Will E. J. , and Davison A. M. , Early Changes in Intracranial Pressure During Haemofiltration Treatment in Patients With Grade 4 Hepatic Encephalopathy and Acute Oliguric Renal Failure, Nephrology Dialysis Transplantation. (1990) 5, no. 3, 192–198, 10.1093/ndt/5.3.192. 2113646

[27] Davenport A. , Continuous Renal Replacement Therapies in Patients With Liver Disease, Seminars in Dialysis. (2009) 22, no. 2, 169–172, 10.1111/j.1525-139X.2008.00539.x. 19426423

[28] Davenport A. and Honore P. M. , Continuous Renal Replacement Therapy Under Special Conditions like Sepsis, Burn, Cardiac Failure, Neurotrauma, and Liver Failure, Seminars in Dialysis. (2021) 34, no. 6, 457–471, 10.1111/sdi.13002. 34448261

[29] Sen B. K. , Pan K. , and Chakravarty A. , Hepatic Encephalopathy: Current Thoughts on Pathophysiology and Management, Current Neurology and Neuroscience Reports. (2025) 25, no. 1, 10.1007/s11910-025-01415-9. 40153081

[30] Churchwell M. D. , Pasko D. A. , Btaiche I. F. , Jain J. C. , and Mueller B. A. , Trace Element Removal During in Vitro and in Vivo Continuous Haemodialysis, Nephrol Dial Transplant Off Publ Eur Dial Transpl Assoc-Eur Ren Assoc. (2007) 22, no. 10, 2970–2977, 10.1093/ndt/gfm352. 17556411

[31] Yurteri G. and Murt A. , Efficiency and Safety of Different Medium Cut-off Membranes in Maintenance Hemodialysis Patients, Seminars in Dialysis. (2026) 39, no. 1-2, 26–32, 10.1111/sdi.70017. 41707698

